# Nitrogen demand and availability: relative roles in driving C_3_ plant responses to elevated CO_2_

**DOI:** 10.1093/jxb/eraf149

**Published:** 2025-07-02

**Authors:** Xuan Hu, Mirindi Eric Dusenge

**Affiliations:** Division of Plant Sciences, Research School of Biology, The Australian National University, Canberra, ACT 2601, Australia; Division of Plant Sciences, Research School of Biology, The Australian National University, Canberra, ACT 2601, Australia

**Keywords:** Acclimation, atmospheric elevated CO_2_, C_3_ plants, eco-evolutionary optimality hypothesis, nitrogen demand, nitrogen availability, photosynthesis, progressive nitrogen limitation hypothesis, *V*_cmax_

## Abstract

This article comments on:

**Perkowski EA, Ezekannagha E, Smith NG.** 2025. Nitrogen demand, availability, and acquisition strategy control plant responses to elevated CO_2_. Journal of Experimental Botany **76**, 2908–2923 https://doi.org/10.1093/jxb/eraf118

This article comments on:

**Perkowski EA, Ezekannagha E, Smith NG.** 2025. Nitrogen demand, availability, and acquisition strategy control plant responses to elevated CO_2_. Journal of Experimental Botany **76**, 2908–2923 https://doi.org/10.1093/jxb/eraf118

**C**_**3**_ **plants generally show increased photosynthesis and whole-plant productivity in response to elevated atmospheric CO**_**2**_ **concentrations [CO**_**2**_ **]. While it is widely recognized that nitrogen (N) mediates the responses of plants to elevated [CO**_**2**_ **], there has been ongoing debate on the relative importance of the N availability versus metabolic N demand in influencing the responses of leaf-level C**_**3**_ **photosynthesis to elevated atmospheric [CO**_**2**_ **]. In this issue of the *Journal of Experimental Botany*, Perkowski *et al*. (2025) provide empirical evidence that N demand strongly dictates the responses of leaf-level photosynthesis while N availability substantially enhances whole-plant productivity in response to elevated [CO**_**2**_ **].**

## Impact of elevated [CO_2_] on C_3_ plants

Atmospheric [CO_2_] has increased by 52% compared with the pre-industrial level (Friedlingstein *et al.*, 2025), and is projected to continue to rise in the near future (Arias *et al*., 2023). Among the main environmental drivers of global change (e.g. CO_2_, temperature, ozone, and drought), elevated atmospheric [CO_2_] and its impact on plants have received relatively more attention. As a result, we have a fairly good understanding of how elevated [CO_2_] affects plants, spanning from the leaf level to the ecosystem scale, and ranging from short-term (minutes) to decade-long studies (Curtis, 1996; Leakey *et al*., 2009; Norby *et al*., 2010; Warren *et al*., 2015). Since plants use CO_2_ as the main substrate during photosynthesis, rising atmospheric [CO_2_] generally increases rates of net photosynthesis on both short -and long-term time scales (Curtis, 1996; Ainsworth and Long, 2005; Leakey *et al*., 2009; Way *et al*., 2015). However, the initial stimulation diminishes over time as plants acclimate to elevated [CO_2_] (Curtis, 1996; Ainsworth and Long, 2005; Leakey *et al*., 2009). At the leaf level, the acclimation of photosynthesis to elevated [CO_2_] involves several changes in physiology and biochemistry (Ainsworth and Long, 2005; Ainsworth and Rogers, 2007; Leakey *et al*., 2009). These changes mainly include decreases in stomatal conductance and the maximum carboxylation rate of Rubisco (*V*_cmax_), which is a key enzyme during the CO_2_ fixation process. The decrease in *V*_cmax_ is driven by an imbalance between the source (leaves) and sink (other parts of the plant), caused by a relatively higher accumulation of sugars in the leaves under elevated [CO_2_]. This accumulation initiates hexokinase signalling pathways, which suppress Rubisco transcription, leading to reduced Rubisco content (Moore *et al*., 1999; Ainsworth and Rogers, 2007), which is reflected in decreases in *V*_cmax_ (Ainsworth and Long, 2005).

## The role of N in C_3_ plant acclimation to elevated [CO_2_]

Photosynthetic acclimation to elevated [CO_2_] has been shown to be stronger in N-poor systems (Ainsworth and Long, 2005). Studies using whole-tree chambers (WTCs) and free air CO_2_ enrichment (FACE) technologies show that initial net primary productivity (NPP) stimulation decreases over time due to N limitation (Oren *et al*., 2001; Luo *et al*., 2004; Norby *et al*., 2010; Warren *et al*., 2015). In a WTC study, elevated [CO_2_] did not significantly affect tree growth at an N-poor site (Sigurdsson *et al*., 2013). Similarly, controlled studies on small plants showed stronger acclimation in plants grown in small pots (<5 litres) or with insufficient fertilization (Sage, 1994; Curtis and Wang, 1998). These findings further highlight the importance of N in driving the long-term acclimation of C_3_ plants to elevated [CO_2_]. The previously observed N-driven reduction in the stimulation of elevated [CO_2_] led to the development of the progressive nitrogen limitation (PNL) framework (Luo *et al*., 2004). The PNL framework suggests that N becomes progressively limiting because it is increasingly stored in the organic matter of additional long-lived plant biomass and soil organic matter, both enhanced by elevated [CO_2_]. Without N fertilization, this process gradually reduces the N available for further plant uptake over time. While the PNL framework explains commonly observed results in N-limited systems quite well, it does not adequately explain the reduction in *V*_cmax_ and associated leaf N commonly observed in elevated [CO_2_] studies with adequate plant soil N availability (Ainsworth and Long, 2005; Dusenge *et al*., 2024). By contrast, the eco-evolutionary optimality (EEO) theoretical framework suggests that the leaf-level demand to build and maintain the photosynthetic machinery under given environmental conditions is the main driver of leaf N content (Harrison *et al*., 2021). The EEO theory proposes that investment in photosynthetic machinery is optimized for prevailing environmental conditions (e.g. light and temperature) in a way that minimizes resource use (N and water), making it less dependent on soil N availability (Smith *et al*., 2019). Therefore, under elevated [CO_2_], plants invest relatively less N to build Rubisco, as it can optimally fix more [CO_2_] under these conditions. Consequently, plants grown under elevated [CO_2_] should exhibit reduced leaf N due to the decreased demand for *V*_cmax_ (Smith and Keenan, 2020), regardless of soil N availability. This is mainly because the Rubisco enzyme accounts for up to 50% of the total leaf N (Spreitzer and Salvucci, 2002).

The new study by Perkowski *et al*. (2025) systematically tested the relative importance of the PNL versus EEO frameworks in the context of plant responses to elevated [CO_2_]. The authors grew soybean plants (*Glycine max*) at nine N fertilization levels under either an ambient atmospheric [CO_2_] of 420 ppm or an elevated atmospheric [CO_2_] of 1000 ppm. Since soybean can fix atmospheric N via its symbiotic relationship with N-fixing bacteria, half of the plants were allowed to fix atmospheric N, while the other half were sterilized to disable this ability, in order to also investigate the role of the N acquisition strategy. As expected from the EEO framework, leaf N overall decreased in plants grown under elevated [CO_2_]. While soil N fertilization increased leaf N, this stimulation was less pronounced under elevated [CO_2_], leading to a greater reduction in leaf N at higher soil N levels compared with ambient [CO_2_]. Similarly, *V*_cmax_ at 25 °C (*V*_cmax25_) decreased by an average of 16% under elevated [CO_2_], and these reductions were not modified by soil N fertilization treatments. Rates of net photosynthesis at prevailing growth [CO_2_] (*A*_growth_) were still higher in elevated [CO_2_] plants compared with their ambient [CO_2_] counterparts. As a result, photosynthetic N use was substantially increased by elevated [CO_2_]. As previously observed in FACE studies (Ainsworth and Long, 2005), total leaf area and total biomass increased under elevated [CO_2_]. While soil N fertilization also increased these traits, the increases were more pronounced under elevated [CO_2_] than in ambient [CO_2_] conditions, as would be expected from the PNL framework. Soil N fertilization had a stronger effect on total leaf area and total biomass in uninoculated compared with inoculated plants. The study by Perkowski *et al*. (2025) also demonstrated that elevated [CO_2_] increased below-ground biomass as a strategy to acquire more N, a pattern that was more pronounced in uninoculated plants. Additionally, increased soil N fertilization reduced the carbon costs to acquire N more significantly in uninoculated than in inoculated plants. Surprisingly, inoculation with symbiotic N-fixing bacteria did not overall influence either leaf-level or whole-plant responses to elevated [CO_2_].

## Implications and future prospects

**Fig. 1.**
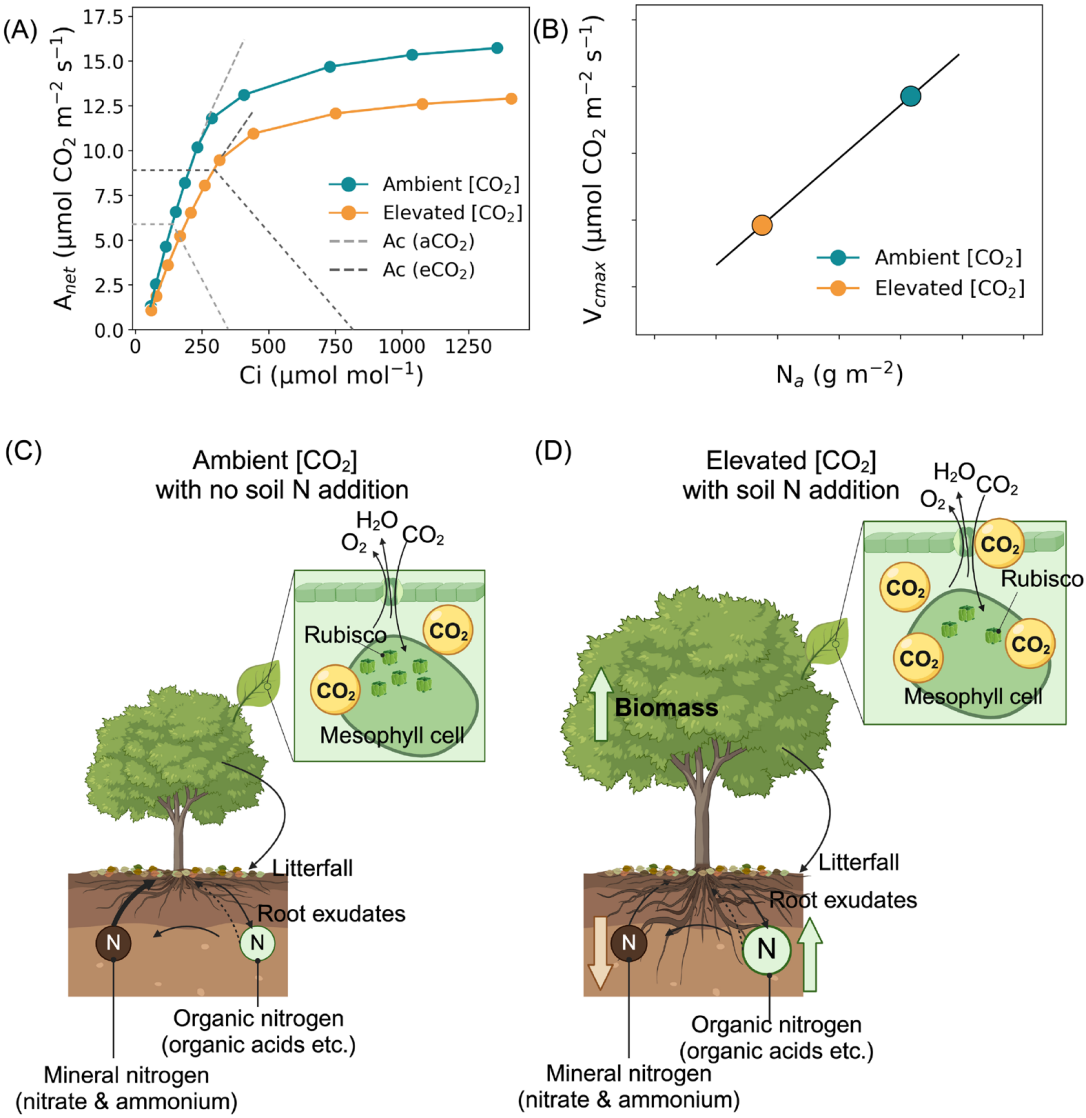
Conceptual representation of C_3_ plant responses to elevated [CO_2_] and soil N availability. (A) Response of net photosynthesis at different air CO_2_ concentrations, also known as the *A*–*C*i curve. The data used to construct this curve are from the study by Dusenge *et al*. (2024), and they focus on black spruce (*Picea mariana*) needles measured at a leaf temperature of 25 °C. In this study, mature black spruce trees were exposed to both ambient atmospheric [CO_2_] and elevated [CO_2_] (ambient+500 ppm) for 1 year. The observed down-regulation of *V*_cmax_ (~19%) is comparable with the 16% observed in Perkowski *et al*. (2025). (B) Decreases in *V*_cmax_ are related to decreases in leaf N (Ainsworth and Long, 2005). (C, D) Schematic representation of the effects of elevated [CO_2_] and high plant-available soil N on the leaf and whole-plant level. At the leaf-level, Rubisco content decreases, regardless of soil N availability, because the leaf can fix more CO_2_ molecules with fewer Rubisco enzymes. At the whole-plant level, exposure to elevated [CO_2_] coupled with high plant soil N availability leads to increased biomass (leaves, stems, and roots), as shown by Perkowski *et al*. (2025). Specifically, under elevated [CO_2_], plants produce more leaves with reduced *V*_cmax_ and N per leaf. These results also highlight how the EEO framework partly acknowledges the importance of plant soil N availability for whole-plant responses, where plants use this extra N to build more optimally coordinated leaves and other plant organs.

The study by Perkowski *et al*. (2025) reconciled the PNL and EEO frameworks by demonstrating that the EEO framework explains leaf-level responses to elevated [CO_2_], while the PNL framework explains whole-plant responses. Specifically, they showed that leaf demand for building a photosynthetic machinery that is adjusted to the plant’s growing environment drives the common enhancement of *A*_growth_ under elevated [CO_2_], despite apparent decreases in photosynthetic capacity (*V*_cmax_) that were unaltered by soil N availability (Fig. 1A, B). The authors found a stronger reduction in leaf N content under elevated [CO_2_] when N was more available, suggesting that plants reallocate N to other non-photosynthetic metabolic processes, ultimately contributing to enhanced plant growth with higher photosynthetic N use efficiency. In contrast, their results show that the PNL framework nicely explains the enhancement of whole-plant responses to elevated [CO_2_] under higher soil N availability, as evidenced by substantial increases in leaf and total biomass (Fig. 1C, D). Findings from this study are consistent with recent results from a field study on mature boreal conifers exposed to both warming and elevated [CO_2_] (Dusenge *et al*., 2024). In a boreal peatland dominated by tamarack and black spruce, 2 years of both below-ground and above-ground warming enhanced plant-available soil N (Iversen *et al*., 2023), which led to an increase in leaf N across the warming treatments. However, despite this increase in leaf N, it did not affect the acclimation of *V*_cmax25_ and leaf N (i.e. decreases) to 1 year of elevated [CO_2_], which remained consistent across all warming treatments (Fig. 1A, B). While these field observations suggest that the results from Perkowski *et al*. (2025) may be applicable to other plant functional types (PFTs), they still need to be tested across a wider range of PFTs, with longer treatment durations, and ideally under field conditions, before any definitive conclusions can be drawn. We also need a deeper understanding of plant N allocation under elevated [CO_2_]. While logistically and financially challenging, these research questions could be integrated into ongoing FACE experiments to improve modelling of the coupling of carbon and N cycles under current and future climatic conditions.
